# A novel dephosphorylation targeting chimera selectively promoting tau removal in tauopathies

**DOI:** 10.1038/s41392-021-00669-2

**Published:** 2021-07-14

**Authors:** Jie Zheng, Na Tian, Fei Liu, Yidian Zhang, Jingfen Su, Yang Gao, Mingmin Deng, Linyu Wei, Jingwang Ye, Honglian Li, Jian-Zhi Wang

**Affiliations:** 1 Department of Pathophysiology, School of Basic Medicine, Key Laboratory of Education Ministry of China/Hubei Province for Neurological Disorders, Tongji Medical College, Huazhong University of Science and Technology, Wuhan, China; 2 Department of Pharmacology, Key Laboratory of Basic Pharmacology of Ministry of Education, Joint International Research Laboratory of Ethnomedicine of Ministry of Education, Key Laboratory of Basic Pharmacology of Guizhou Province, Zunyi Medical University, Zunyi, China; 3 Department of Histology and Embryology, Key Laboratory of Ministry of Education for Neurological Disorders, School of Basic Medicine, Tongji Medical College, Huazhong University of Science and Technology, Wuhan, China; 4 Co-innovation Center of Neuroregeneration, Nantong University, Nantong, China

**Keywords:** Drug development, Neurological disorders

## Abstract

Intraneuronal accumulation of hyperphosphorylated tau is a hallmark pathology shown in over twenty neurodegenerative disorders, collectively termed as tauopathies, including the most common Alzheimer’s disease (AD). Therefore, selectively removing or reducing hyperphosphorylated tau is promising for therapies of AD and other tauopathies. Here, we designed and synthesized a novel DEPhosphorylation TArgeting Chimera (DEPTAC) to specifically facilitate the binding of tau to Bα-subunit-containing protein phosphatase 2A (PP2A-Bα), the most active tau phosphatase in the brain. The DEPTAC exhibited high efficiency in dephosphorylating tau at multiple AD-associated sites and preventing tau accumulation both in vitro and in vivo. Further studies revealed that DEPTAC significantly improved microtubule assembly, neurite plasticity, and hippocampus-dependent learning and memory in transgenic mice with inducible overexpression of truncated and neurotoxic human tau N368. Our data provide a strategy for selective removal of the hyperphosphorylated tau, which sheds new light for the targeted therapy of AD and related-tauopathies.

## Introduction

Intraneuronal accumulation of the neurofibrillary tangles formed primarily by hyperphosphorylated tau is a hallmark of a collection of neurodegenerative disease named tauopathies, including Alzheimer’ disease (AD),^1,2^ the most common form of dementia in the elderly. Recently, multiple efforts are being made to reduce the tau accumulation in AD, by using such as tau antisense oligonucleotides,^3^ antibodies,^4^ PROteolysis TArgeting Chimeras (PROTACs),^5,6^ and the structure-based tau aggregation inhibitors.^7,8^ It is well known that tau hyperphosphorylation initiates the abnormal tau aggregation and induces neurodegeneration in the brains of AD and the related tauopathies,^9^ however, there is currently no strategy for specifically targeting tau phosphorylation at the post-translational level.

Inhibiting serine/threonine protein kinases, such as glycogen synthase kinase-3β (GSK-3β), cyclin-dependent kinase-5 (CDK-5), and extracellular signal-regulated kinase 1/2 (ERK1/2), can ameliorate tau hyperphosphorylation and pathologies in AD.^1^ However, kinases inhibitors showed poor selectivity in reducing tau phosphorylation. An alternative strategy to inhibit tau hyperphosphorylation is to strengthen tau phosphatases. Among the currently identified tau phosphatases, protein phosphatase 2A (PP2A) is most active in dephosphorylating tau protein both in vitro and in vivo.^10^ The heterotrimeric PP2A holoenzyme is consisted of a scaffold subunit A, a regulatory subunit B (recognizing and targeting substrates) and a catalytic subunit C. There are many PP2A subfamilies, among which the Bα-containing isoform exhibits the highest efficiency in dephosphorylating tau.^11^ Given the loss-of-function of PP2A in AD brains,^12,13^ enhancing PP2A activity by drugs should be a promising way for preventing the AD-related tau hyperphosphorylation, while it should be noted that nonselective pharmacological PP2A activators may induce unendurable side-effects, because PP2A is a key regulator involved in multiple cellular metabolic processes.^14^

Inspired by the tau PROTACs that can specifically recruit E3 ligase or proteolysis-relevant chaperones to degrade tau roughly,^5,6^ we designed and synthesized here a novel DEPhosphorylation TArgeting Chimaera (termed as DEPTAC) to selectively facilitate the link between tau and PP2A-Bα. By using the DEPTAC, a specific and efficient clearance of the AD-like hyperphosphorylated tau has been achieved both in vitro and in vivo.

## Results

### DEPTAC binds to tau and PP2A with high neuronal penetrability

**Fig. 1:**
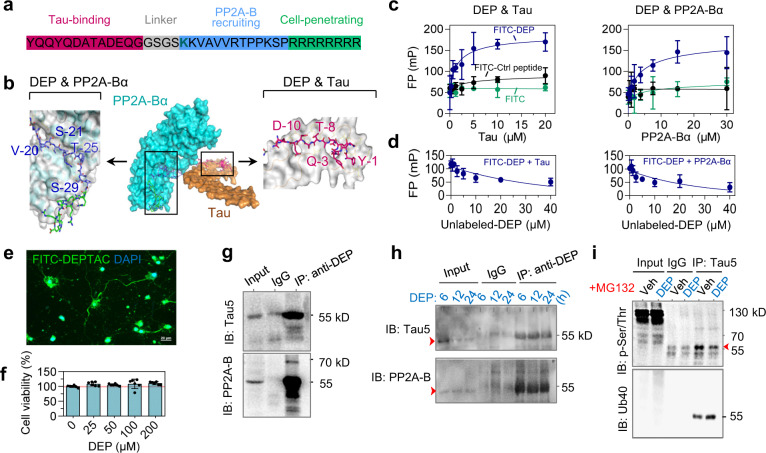
DEPTAC binds to tau and simultaneously recruits PP2A-Bα. **a** Functional motifs and amino acid sequence of DEPTAC (DEP). **b** The estimated triadic interaction of DEPTAC with tau (khaki) and PP2A-Bα (cyan) based on their 3D-structures in ZDOCK Server. Key binding residues of DEPTAC were as labeled. **c**, **d** Direct interactions of FITC-conjugated DEPTAC to tau and PP2A-Bα measured by fluorescence polarization binding (**c**) and the competing (**d**) assays. **e** High cell penetrability of DEPTAC in cultured primary rat neurons. Scale bar, 20 μm. **f** No cytotoxicity of the DEPTAC (to 200 μM) detected by CCK8 assay. Data were normalized by the mean value of the 0-μM group. One-way ANOVA followed by Tukey’s multiple comparisons tests. *F*(4, 25) = 1.47, *P* = 0.24. **g** The triadic interaction of DEPTAC with tau and PP2A-B was confirmed by immunoprecipitation using DEPTAC antibody in primary cultured rat neurons (12 h). **h** Co-immunoprecipitation of DEPTAC with tau and PP2A-B can be detected 6–24 h of post-DEPTAC treatment, with a gradual decrease of total tau. **i** DEPTAC decreased tau phosphorylation at Ser/Thr residues with upregulated tau ubiquitination in the presence of MG132

The DEPTAC (~4.4 kDa, C_185_H_314_N_72_O_56_) consists 38 amino acid residues from N-terminals to C-terminals and has four functional motifs (Fig. 1a): (1) YQQYQDATADEQG, originates from the porcine β-tubulin 422–434 for recognizing and binding tau proteins.^5,6^ (2) GSGS, a linker for increasing the flexibility of the peptide.^15^ (3) KKVAVVRTPPKSP, for recruiting PP2A-Bα,^16^ and (4) RRRRRRRR, for cell penetrating.^17^ A peptide with mutations in both the tau-binding and PP2A-Bα recruiting domains (YQQYQAATAAAQG-GSGS-KAVAVVRTPPASP-RRRRRRRR) was used as a control. For tracing the chimera, a fluorescein FITC was conjugated at the K18 residue.

DEPTAC is designed to selectively bind tau and simultaneously recruit PP2A-Bα. The bindings of DEPTAC with tau and PP2A-Bα were showed in the ZDOCK Server based on their 3D-structures (Fig. 1b), and confirmed in vitro by fluorescence polarization binding and competing assays, in which sigmoidal increases of FITC-DEPTAC fluorescence polarization were shown with increased amount of tau and PP2A-Bα (Fig. 1c), respectively, and both were reversed by increased amount of the unlabeled-DEPTACs (Fig. 1d).

The DEPTAC showed high neuron penetrability both in vitro and in vivo (Fig. 1e and Supplementary Fig. S1a), low cytotoxicity in primary culture (Fig. 1f), but limited brain-blood barrier (BBB) penetrating efficiency when delivered through mouse tail vein (Supplementary Fig. S1b).

To further test the triadic interaction of DEPTAC with tau and PP2A-Bα, we developed a rabbit polyclonal antibody of DEPTAC using a fragment of DEQGGSGSKKVA (Supplementary Fig. S2). The co-immunoprecipitation of DEPTAC with tau and PP2A-B were detected in cultured primary neurons 24 h post DEPTAC treatment (Fig. 1g), with a time-dependent decrease of total tau (Fig. 1h), which might be attributed to the facilitated dephosphorylation of tau that precedes and facilitates its proteolysis.^18–21^ This idea was confirmed by the downregulation of tau phosphorylation and upregulation of tau ubiquitination by DEPTAC when we used MG132, a proteasome inhibitor, to hinder the reduction of tau (Fig. 1i).

### DEPTAC facilitates tau dephosphorylation and degradation in cultured neurons

**Fig. 2:**
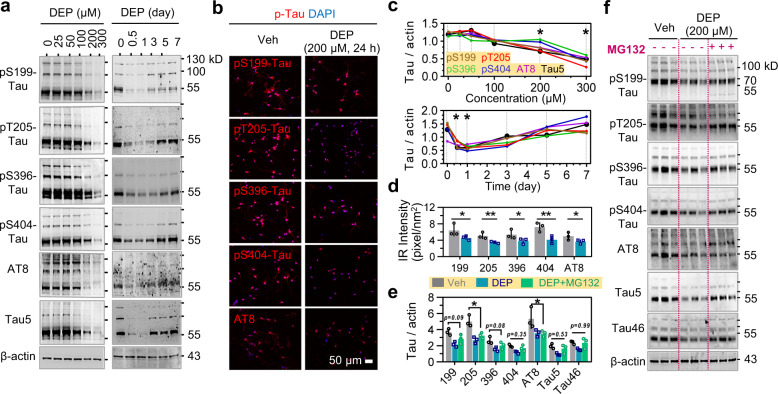
DEPTAC facilitates tau dephosphorylation and degradation in primary neurons with hTau-overexpression. **a**–**d** DEPTAC promoted tau dephosphorylation at the AD-related pS199, pT205, pS396, pS404, and AT8 epitope with reduced total tau probed by Tau5 antibody, in a dose- and time-dependent manner in cultured primary rat neurons with lentivirus-mediated hTau-overexpression (**a**, **c**). Data were normalized to the level of β-actin. One-way ANOVA followed by Tukey’s multiple comparisons tests, *p < 0.01 compared with baseline, *n* = 3 cell wells for each group. By immunofluorescence staining, DEPTAC (200 μM for 24 h) also reduced the averaged immunoreactive (IR) intensity of phospho-tau (**b**, **d**). Scale bar, 50 μm. Two-way ANOVA followed by Tukey’s multiple comparisons tests. *p < 0.05, **p < 0.01, *n* = 3 cell wells in each group. **e**, **f** DEPTAC promoted tau dephosphorylation with reduced efficiency in the presence of MG132. Two-way ANOVA followed by Tukey’s multiple comparisons tests, *p < 0.05, *n* = 3 cell wells in each group

We next examined the efficiency of DEPTAC in reducing tau phosphorylation at multiple AD-related sites, including the Ser199, Thr205, Ser396, Ser404, and the AT8 (Ser199/Ser202/Thr205) epitopes,^11^ in primary cultured rat hippocampal neurons with lentivirus-mediated overexpression of human tau (hTau). DEPTAC downregulated tau phosphorylation at all those sites, and the most significant reduction of the phospho-tau was shown by DEPTAC at about 200–300 μM and within 3 days post administration (Fig. 2a–d). Again, we also observed here a reduction of total tau probed by Tau5 antibody in addition to the reduction of phospho-tau (Fig. 2a–d). DEPTAC did not change the level of MAP2, another microtubule-associated protein (Supplementary Fig. S3), which confirms the specificity of DEPTAC to tau.

To test whether the DEPTAC-mediated tau dephosphorylation was dependent on the reduction of total tau, we used MG132 to prevent the proteolysis of tau following DEPTAC administration. The reduction of total tau detected by Tau5 and Tau46 antibodies was abolished in the presence of MG132, but notably, the DEPTAC-induced tau dephosphorylation was still significant, especially at pT205 and AT8 epitope though with less efficiency (Fig. 2e, f). Indeed, it is known that tau dephosphorylation is generally required and facilitates its proteolysis,^20,21^ while tau hyperphosphorylation hinders its degradation.^18,19,22^ Our data indicate that the reduction of p-tau by DEPTAC is not dependent on but mutually facilitated by the proteolysis of tau. The reduced dephosphorylating effectiveness of DEPTAC by MG132 might be due to the dysregulated PP2A activity^23,24^ or the hindered recycling of DEPTAC. In general, the DEPTAC-mediated clearance of both phosphorylated tau and total tau could finally benefit the amelioration of tau pathologies in AD.^1^

By contrast to neurons, we observed that DEPTAC showed relatively limited effectiveness in dephosphorylating and reducing tau in hTau-expressed HEK293 cells (Supplementary Fig. S4). This discrepancy might be due to the very much different cell metabolic processes and/or Bα-containing PP2A sufficiency in HEK293 cell, a cell line derived from human embryonic kidney cells sharing lots of differences in transcriptome with neuron. Interestingly, the neuron-specific high efficiency is an unexpected benefit for AD drug development. Thus, we mainly focused on the neurons in the present study.

### DEPTAC ameliorates phospho-tau accumulation in human tau transgenic mice

We next tested whether DEPTAC can reduce phospho-tau in vivo. In 9-month 3×Tg AD mice, 5 mM DEPTAC significantly reduced phospho-tau and total tau levels in the hippocampus when repeatedly delivered for a consecutive month (once every 3 days, 1 μL each), though the effectiveness was relatively limited when administrated for once (Supplementary Fig. S5).

**Fig. 3:**
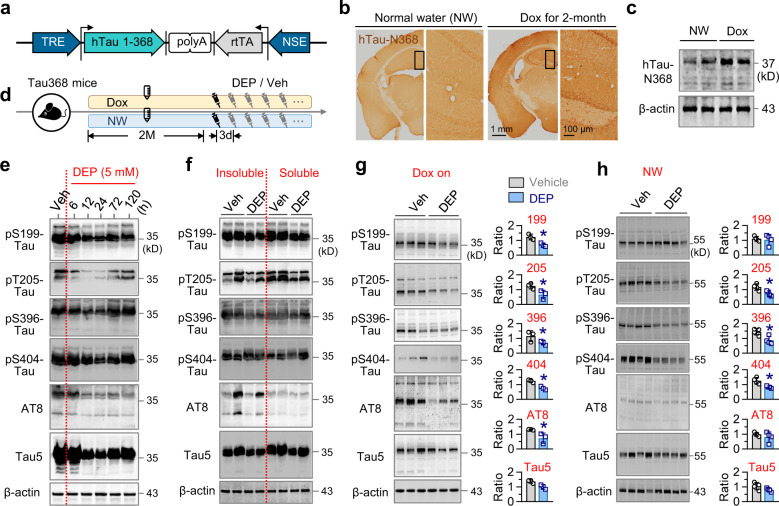
DEPTAC reduces phosphorylated and total tau in the hippocampus of Tau368 mice. **a** The expression of hTau-N368 was controlled by the NSE promoter and a tet-on system. **b**, **c** Prominent hTau-N368 expression was detected by immunohistochemistry (**b**) and Western blotting (**c**) in the dorsal hippocampus of the dox-, but not normal water (NW)-drinking group. **d** Experimental procedures for drugs administration. DEPTAC or vehicle was administrated for once or a consecutive month during dox on. **e** DEPTAC for once reduced phosphorylated and total tau prominently at 5 mM and 12–72 h of post intra-cerebroventricular injection in the hippocampus of Tau368 mice. **f** DEPTAC decreased both soluble and insoluble tau in the hippocampus of Tau368 mice. **g**, **h** DEPTAC for a consecutive significantly reduced hippocampal phospho-tau and total tau (/ 10 μg total protein) after dox administration for 4 months (**g**), while the efficiency of DEPTAC on mouse tau was relatively limited in NW-administrated Tau368 mice (/ 30 μg total protein) (**h**). Unpaired *t*-tests, *p < 0.05, *n* = 3–4 mice in each group

To avoid the potential disturbing of tau phosphorylation and degradation by *APP* and *PSEN1* genes mutations, and the impact of human tau expression on murine embryonic development in 3 × Tg AD mice,^25,26^ we tested the efficiency of DEPTAC in a self-developed transgenic mouse line named Tau368 mice, in which the neurotoxic^27^ human tau N-terminal 1–368 fragment (hTau-N368) is overexpressed under the control of neuron-specific enolase (NSE) promoter together with a tet-on system (Fig. 3a). Doxycycline (dox) was administrated through drinking water (2 mg/mL) and induced abundant hTau-N368 expression in the hippocampus 2 months post dox administration (Fig. 3b, c and supplementary Fig. S6). Tau368 mice treated with normal water (NW), which share same genetic background with the those treated with dox but show no hTau-N368 expression, were used as control. We found that 5 mM DEPTAC treatment for a consecutive month (once every 3 days, 1 μL each) was effective in reducing phosphorylated and total tau in the hippocampus of dox-treated Tau368 mice generally 12–72 h post cerebroventricular injection (Fig. 3d, e), and both radioimmunoprecipitation assay (RIPA) lysis buffer-soluble and insoluble tau tend to be decreased 24 h of post-DEPTAC administration (Fig. 3f).

We next tested the chronic effect of DEPTAC on tau when hTau-N368 was continuously expressed. Dox was kept on for 3 months, and DEPTAC was repeatedly delivered through a guiding cannula implanted into the lateral ventricle of Tau368 mice (once every three days, for a consecutive month) before detection. Still, we found that DEPTAC effectively facilitated the dephosphorylation as well as the degradation of tau (Fig. 3g).

In control mice (i.e., Tau368 mice with only NW but no dox-induction), the phosphorylation of tau was nearly undetectable by the same protein loading as dox-groups (10 μg, data not shown). By increasing protein loading (30 μg), we detected tau phosphorylation at multiple sites in varying degrees, and found that DEPTAC could also significantly downregulate the phosphorylation level of endogenous mouse tau at Thr205, Ser396, and Ser404 but showed limited effect on Ser199, AT8, and the total tau (Fig. 3h). These data together suggest a relatively limited side-effect of DEPTAC on tau under normal conditions.

### DEPTAC improves neurite plasticity and microtubule assembly

**Fig. 4:**
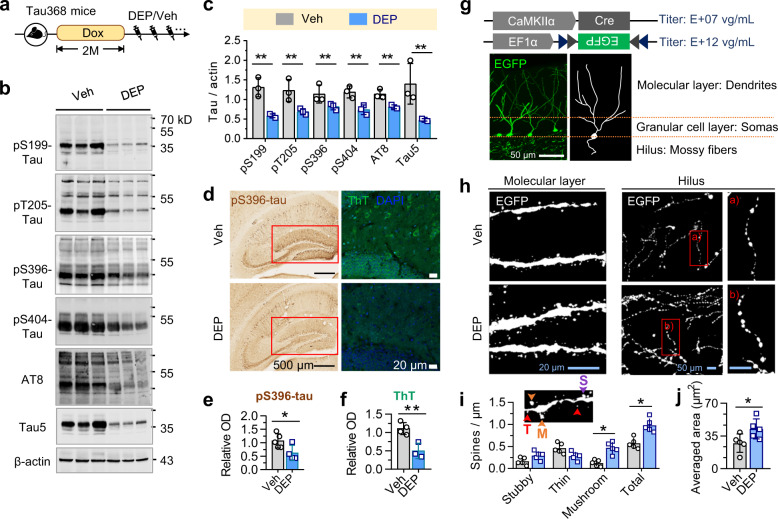
DEPTAC promotes tau dephosphorylation and improves neurite plasticity in dox-treated Tau368 mice. **a** Experimental procedure showing that DEPTAC or vehicle was administrated for a consecutive month after stopping the dox. **b**, **c** DEPTAC downregulated phospho-tau and total tau levels in the hippocampi of Tau368 mice. Two-way ANOVA followed by Tukey’s multiple comparisons tests. **p < 0.01, *n* = 3 mice in each group. **d**–**f** DEPTAC ameliorated the level of pS396-tau immunostaining (**e**) and thioflavin T (ThT)-stained tau aggregation (**f**) in the DG subset (red boxes). Unpaired *t*-tests, **p < 0.01, *n* = 4–5 mice in each group. (**g**) Sparse labeling of dentate granular cells by a mixture of AAVs. **h**–**j** DEPTAC increased the densities of overall and the mushroom-like dendritic spines (**i**) and averaged area of mossy fiber puncta (**j**) of granular cells. The insert (**i**) illustrated the morphology of different types of dendrite spines: T thin, M mushroom-like, S stubby. Two-way ANOVA followed by Tukey’s multiple comparisons tests or unpaired *t*-tests, *p < 0.05, *n* = 5 mice in each group

We next evaluated the effect of DEPTAC in ameliorating tau-related hippocampal pathologies in the hTau mice. DEPTAC was administrated for a consecutive month after stopping the dox-induced hTau-N368 expression to potentially maximize the efficiency of tau dephosphorylation (Fig. 4a). Prominently, repeated DEPTAC administration reduced both phospho-tau and total tau levels in the hippocampi of Tau368 mice (Fig. 4b, c), especially in the dentate gyrus (DG) subset (Fig. 4d, e). Besides, tau aggregation in the DG shown by thioflavin T staining was also ameliorated by DEPTAC (Fig. 4d, f).

Abnormal accumulation of phospho-tau hinders neurite differentiation and synapse formation.^28^ To evaluate how DEPTAC affects neurites morphology, we co-infused a mixture (1:1 vol/vol) of AAV-CaMKIIα-Cre (titer 5.21E + 07 vg/mL) and AAV-EF1α-DIO-EGFP (titer 3.24E + 12 vg/mL) into the dorsal hippocampal DG of Tau368 mice to sparsely label excitatory neurons (Fig. 4g). Mice with only granular cells labeled were included in analysis. DEPTAC significantly increased the mushroom-like and total dendritic spine densities, and enlarged mossy fiber puncta areas (Fig. 4h–j), indicating an improvement of neurite plasticity by DEPTAC in Tau368 mice. By contrast, only limited effects of DEPTAC on the spine density and mossy fiber puncta areas were detected in Tau368 mice without dox administration (Supplementary Fig. S7). By Sholl analysis, no statistically significant change in dendrite complexity was detected by DEPTAC in both dox-treated and NW-treated mice (Supplementary Fig. S8).

**Fig. 5:**
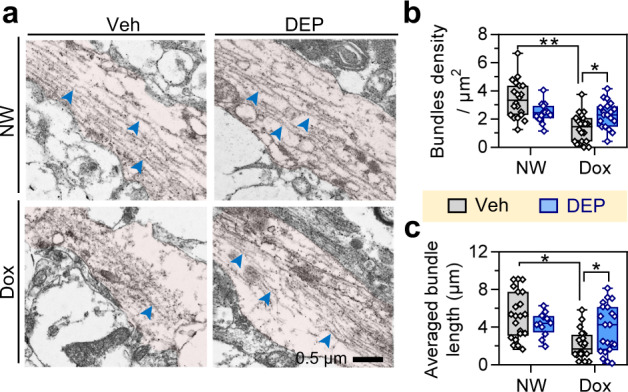
DEPTAC improves microtubule assembly in dox-treated Tau368 mice. **a** Representative electron microscopy images. Pink areas indicated the neurites of hippocampal neurons, and the blue arrowheads indicate the representative polymerized microtubules. Scale bar, 500 nm. **b**, **c** Dox for 2 months decreased the density (**b**) and length (**c**) of microtubule bundles in Tau368 mice, while both of which were significantly reversed by 1-month DEPTAC treatment after stopping the dox. Two-way ANOVA followed by Tukey’s multiple comparisons tests, *p < 0.05, *n* = 5 mice and 12–23 view fields (circles) in each group. Data were presented as Min-Median-Max

Intracellular hyperphospho-tau accumulation has been also well evidenced to disrupt microtubule assembly and stability.^29^ We found here that the hTau-N368 overexpression (dox for 2 months) resulted in prominent microtubule depolymerization within the neurites of the hippocampal neurons, while DEPTAC treatment for a consecutive month followed by stopping dox administration (Fig. 4a) efficiently reversed the microtubule disassembly in morphology (Fig. 5).

These data together indicate that DEPTAC can improve neurite plasticity and facilitate microtubule assembly in tauopathies.

### DEPTAC improves hippocampus-dependent cognitive impairments in Tau368 mice

**Fig. 6:**
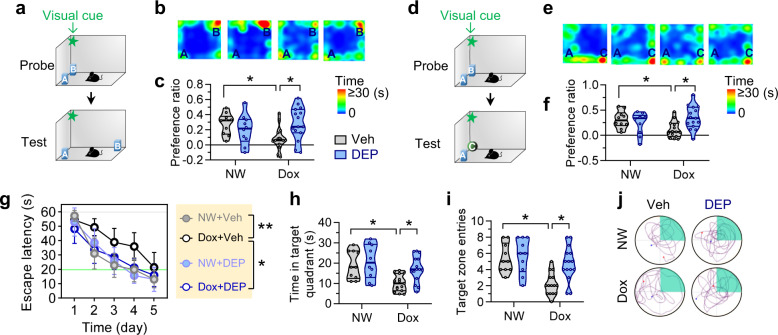
DEPTAC improves hippocampus-dependent learning and memory in dox-treated Tau368 mice. **a**–**f** 1-month DEPTAC treatment after stopping the dox rescued the hTau-induced object-place (**a**–**c**) and novel-object (**d**–**f**) recognition impairments in Tau368 mice compared with the vehicle controls. Cartoons (**a**, **d**) show paradigms of the behavioral tests. Representative heatmaps (**b**, **e**) show the time-location of mice traveled in test chambers. Two-way ANOVA followed by Tukey’s multiple comparisons tests, *p < 0.05, *n* = 10–15 mice in each group. **g** DEPTAC significantly alleviated the hTau-induced spatial learning deficits in Morris water maze test. Repeated measures ANOVA followed by Tukey’s multiple comparisons tests, **p < 0.01, *p < 0.05. **h**, **i** DEPTAC attenuated spatial memory deficits in dox-treated Tau368 mice shown by the increased time staying in **h** and number of entries into (**i**) the target quadrant in water maze test detected at day 6 after removed the platform. Two-way ANOVA followed by Tukey’s multiple comparisons, *p < 0.05. **j** Representative traces of the mice traveled in the water maze at day 6. Green areas indicate the target quadrants. Data were presented as Min-Median-Max

Lastly, we examined how DEPTAC affects hippocampus-dependent learning and memory. The dox-induced overexpression of hTau-N368 induced cognitive impairments in discriminating objects moved to the novel places (Fig. 6a–c) and recognizing novel objects (Fig. 6d–f), while repeated DEPTAC administration for a consecutive month (followed by stopping the dox) remarkably ameliorated those cognitive deficits. Consistently, DEPTAC also rescued the spatial learning (Fig. 6g) and memory (Fig. 6h–j) impairments in dox-Tau368 mice measured by Morris water maze (MWM) test. Meanwhile, DEPTAC only limitedly affected the cognitive functions in control mice treated with NW (Fig. 6).

## Discussion

Tau hyperphosphorylation initiates most tauopathies including AD.^9^ Intracellular accumulation of hyperphosphorylated tau gives rise to neurofibrillary tangles, dysregulates neuronal excitability,^30^ impairs synaptic plasticity, disrupts interneuronal transmission^31^ thus induces learning and memory impairments. As a large number of new drugs for β-amyloid clearance in AD showed limited efficiency in halting disease progression in clinical trials, tau protein attracts increasing attentions as a new therapeutic target.^32^ For instance, developing drugs to prevent tau translation from mRNA by antisense nucleotides,^3^ and to promote tau degradation with PROTACs^5,6,33^ are being carried on. Given that abnormal tau accumulation is generally initiated by its hyperphosphorylation,^9^ we developed in the present study a novel tau-based therapeutic tool by specifically facilitating tau dephosphorylation at the post-translational level, which might provide an early-stage treatment of AD and related tauopathies before the onset of abnormal tau accumulation.

The DEPTAC is designed to target tau protein but not its gene expression, since no tau gene mutation has been linked to the AD pathology to date. To maximally replicate the AD-like tau pathologies, we overexpressed full-length wildtype tau (which we have well-evidenced effective in inducing AD-like tau hyperphosphorylation and accumulation^34^), and the more toxic truncated hTau-N368 (derived during aging or pathological conditions^27^) for the in vitro and in vivo studies, respectively. Although tau overexpression may not be the main mechanism for AD, it is well recognized that intraneuronal accumulation of the hyperphosphorylated tau is one of the two hallmarks in the AD brains,^35^ and mediates the synaptic toxicity of Aβ.^26^ Besides, there are over 20 kinds of tauopathies, including frontotemporal dementia, progressive supranuclear palsy, corticobasal degeneration, and so on, showing common abnormalities in tau accumulation in addition to AD. Therefore, we believe that the DEPTAC developed in the current study has great therapeutic potentials for the tau-related neurodegenerative diseases by dephosphorylating tau, thuspreventing tau aggregation and tangle formation.

We found here that DEPTAC could dephosphorylate phosphor-tau at multiple sites with improvements at neuronal morphology and function levels. In fact, the full-length tau protein possesses over 80 Ser-phosphorylation and Thr-phosphorylation sites. Studies suggest that phosphorylation at Thr-205 plays a neuroprotective role,^36,37^ while at Ser-199, Ser-396, and Ser-404 show neurotoxicity.^38,39^ Currently, how these multisite phosphorylation is regulated at different conditions, and how different combinations of the multisite tau phosphorylation determine the overall outcome of tau are challenging issues to be elucidated. Based on the current understanding on tau phosphorylation sites and the functions, it is hard to reconcile the discrepancy of different tau residues phosphorylation. Site-specific chimeras selectively acting on neurotoxic tau phosphorylation epitopes deserves further investigation.

The DEPTAC not only promoted tau dephosphorylation but also facilitated tau degradation. It is well established that the hyperphosphorylated tau proteins are resisted to calpain-induced proteolysis;^18^ the mutual stimulation of tau hyperphosphorylation and SUMOylation inhibits tau degradation with the mechanisms involving a decreased ubiquitination;^19^ Consistently, tau dephosphorylation precedes and is required for its proteolysis.^20,21^ Thus, we think that it should be virtually impossible for DEPTAC to promote tau dephosphorylation without affecting its degradation. Nevertheless, both the reduction of p-tau and total tau are beneficial for tauopathies.^1^

As a microtubule-associated protein, the main function of tau is to promote microtubule assembly and maintain stability of the microtubules. Evidences from tau knock-out and knock-down mice suggest that tau proteins are also involved in synaptic plasticity,^40,41^ iron metabolism,^42^ neuronal maturation,^43^ and ribosomal DNA stability.^44^ However, in addition to the fact that decreasing both p-tau and total tau could be anyhow beneficial for AD treatment,^1^ we think that the potential side-effect from the tau reduction by DEPTAC should be acceptable based on the following facts: (1) tau knockout mice are viable, fertile, immunohistochemically normal in central nervous system in adulthood^45^ and performed indistinguishably from wild-type mice until 6 months of age.^42,46^ (2) It is suggested that the functions of tau may be compensated by other microtubule-associated proteins such as high molecular weight MAPs.^47^ (3) The degree of tau reduction by DEPTAC is far less than tau knock-out or siRNA-mediated knock-down. Indeed, our data showed that DEPTAC did not statistically change the neurite differentiation and spatial learning and memory in control mice without human tau accumulation, which confirm that DEPTAC does not affect the neuronal structure and the function under normal condition.

One of the major advantages for the DEPTAC strategy is that it possesses considerable selectivity to tau proteins. By contrast, small-molecular kinase inhibitors or phosphatase enhancers generally lack specificity. For example, lithium has been used to suppress tau hyperphosphorylation but it also acts on other targets such as the inositol monophosphatase and glycogen synthase kinase-3.^48^ This might bring unacceptable side-effects in the clinic. Importantly, the principle of DEPTAC can be used in future studies to design alternative chimeras specifically targeting many other types of post-translational but pathological tau modifications, such as acetylation,^49,50^ SUMOylation,^19^ glycosylation,^51^ etc. For example, replacing the PP2A-Bα-binding motif in the DEPTAC with sequences for recruiting specific deacetylase or isopeptidase, if functions, might also achieve beneficial effects in ameliorating tau pathologies.

It should be noted that limitations still exist for applying the current DEPTAC as drugs. Although the DEPTAC can be recurrently recruited following tau degradation in theory, a common disadvantage for peptide chimeras is that they only function in efficiency within a short concentration and time windows. We observed here that the DEPTAC was only effective >200 μM and within 3 days of post-administration. The narrow effective window of DEPTAC might be due to the fact that the peptide is often susceptible to the proteolysis. Therefore, it should be taken into consideration when designing new chimeras to improve the peptide stability by, for example, introducing amino acid modifications, D-type amino-acids^7^, or cyclic structures into peptides. Besides, the current DEPTAC is made of 39 amino acids, namely the molecular weight may be too high to penetrate the brain-blood barrier. Thus, the peptide length of DEPTAC could be shorten (under the premise of preserving its selectivity to tau) to facilitate its clinical application in future studies. Moreover, developing new chimeras of universal utility for various tau and PP2A isoforms could further enhance their therapeutic potentials.

Taken together, we developed here a novel chimera termed as DEPTAC that selectively facilitated the recruiting of PP2A to tau, which promoted tau dephosphorylation both in vitro and in vivo. Our innovated DEPTAC provides a novel tool for tau-based drug development in AD and the related tauopathies.

## Materials and methods

### Peptides

DEPTAC and control peptide were synthesized by Haode Peptide (Wuhan, China). For fluorescein-labeling, FITC was conjugated at the K18 residue of DEPTTAC or control peptide. The purity of peptide was evaluated by mass spectrometry and ensured to be ≥95%. All peptides were dissolved in sterile water or phosphate buffer saline (PBS) immediately before administration.

### Viruses and reagents

Lentivirus LV-EF1α-hTau-GFP (3.50E+8 vg/mL) was packaged by OBiO (Shanghai, China) and used for hTau-overexpression (fused with GFP) in cultured primary rat neurons. AAV-CaMKIIα-Cre (diluted to 5.21E + 07 vg/mL) and AAV-EF1α-DIO-mCherry (3.24E + 12 vg/mL) were obtained from BrainVTA (Wuhan, China) and co-injected (1:1 vol/vol) for sparse labeling of dorsal DG neurons. Doxycycline was purchased from Energy Chemical (Shanghai, China). Rabbit polyclonal DEPTAC antibody was prepared by Daian Biotechnology (Wuhan, China). All other reagents including MG132 were purchased from Sigma-Aldrich (USA), unless otherwise specified.

### Animals

Tau368 transgenic mouse was produced by Nanjing Biomedical Research Institute of Nanjing University. Briefly, a fragment of *Insulator-pTRE3G-Kozak-ATG-hMapt (N368) CDS-TGA-polyA-polyA-TAA-rtTA3G-Kozak-ATG-Rat NSE promoter-Insulator* (Fig. 3a) was transferred into mouse fertilized eggs through microinjections, and then eggs were engrafted into pregnant C57BL/6J mice. Offsprings with successful gene transfection were identified through genotyping. Doxycycline (2 mg/mL) was administrated through drinking water to induce hTau-N368 expression from 2-month of age. 3×Tg AD (129S4.Cg-Tg(APPSwe,tauP301L)1Lfa Psen1^tm1Mpm^/LfaJ) mice were purchased from Jackson Laboratory. Male C57BL/6 mice of 2-month age were purchased from Charles River (Weitonglihua, Beijing, China). Pregnant female SD rats (P12–15 days) were obtained from the Experimental Animal Center of Huazhong University of Science and Technology. All mice were kept under standard laboratory conditions, with a 12 h alternating light/dark cycle, food, and water available ad libitum. The influence of sex was not evaluated in this study. For mice, only males weighing 20–30 g were used in all the experiments. All animal experiments were approved by the Animal Care and Use Committee of Huazhong University of Science and Technology.

### Cell culture

Primary hippocampal neurons were prepared from E12–E15 days SD rat embryos. Briefly, pregnant rats were acutely executed, hippocampus was dissected on ice and gently minced in DME/F12 (Hyclone) then suspended in 0.125% (vol/vol) trypsin solution at 37 °C for 20 min. Enzymatic activity was stopped by fetal bovine serum. Neurons were initially kept in 10% FBS-containing DME/F12 for 4–6 h, and subsequently cultured in Neurobasal (Hyclone) with 2% B27, 1% glutamax and 1% penicillin–streptomycin (Thermo Fisher Scientific) in dishes pre-coated with 100 μg/mL poly-d-lysine. Serum was gradually removed by replacing half of the medium for each time within the following 2 days. Especially, to inhibit the proteasome-dependent proteolysis, the cultured hippocampal neurons were treated with 5 μM MG132 for 5 h before harvest.

HEK293 cells with stable expression of wild-type full-length human tau (term as HEK293(hTau) cells), were cultured in DMEM (Hyclone, SH30022.01) containing 10% fetal bovine serum (Biological Industries, 04–001-1ACS) and 200 mg/mL G418 (Thermo Fisher Scientific, 10,131,027) under 5% CO_2_, 37 °C.

### ZDOCK

The linear structure of DEPTAC was simulated by Tleap plugin of Amber12 using the force field 03 (FF03), and its 3D-sturcture was predicted by superposing the crystal structure of tau-recognizing domain from β-tubulin 422–434 (the b-chain of 5NQU) and PP2A-Bα-recruiting domain from Mapt 195–207 (the a-chain of 1I8H), respectively, onto the linear structure of DEPTAC, and then optimized using the sybyl-X2.1 based on molecular dynamics (duration 1000 fs, step size 1 fs, temperature 300 K, force field was Tripos). The binding between DEPTAC with tau (the a-chain of 6NWP) or PP2A-Bα (b-chain of 3FGA) was evaluated, respectively, in the ZDOCK Server (3.0.2) (http://zdock.umassmed.edu/). Key amino acid residues mediating the DEPTAC-protein binding of patterns with the highest binding score were estimated.

### Fluorescence polarization assay

Purified human tau and PP2A-Bα (PPP2R1A) were obtained from AtaGenix (Wuhan, China), and concentrated using ultrafiltration tubes (Beyotime). In basic binding assays, tau or PP2A-Bα was aliquoted into serial dilutions with a buffer containing 20 mM Tris-HCl, 150 mM NaCl, and 0.5 mM 1,4-Dithiothreitol (pH 7.4), and incubated with 0.1 μM FITC-labeled DEPTAC, FITC-labeled control peptide and FITC (9:1 vol/vol), respectively, in a black 384-well plates (Corning) under 37 °C for 30 min. In competition binding assays, a mixture of 2.5 μM tau or PP2A-Bα and 0.1 μM FITC-labeled DEPTAC were incubated with increasing amounts of unlabeled-DEPTAC under 37 °C for 30 min. Fluorescence intensity was measured by a Biotek Synergy 2 microplate reader with the wavelengths of 480 nm (60) (Ex)/560 nm (40) (Em). The polarization measurements were corrected for background contributions to the measured intensity by subtracting the parallel and perpendicular intensity readings by the vehicle-only wells from the intensity readings for each data point. Fluorescence polarization (*P*) was calculated by the formula P = (*I*_║_ − *I*_┴_) / (*I*_║_ + *I*_┴_), where *I*_║_ was the intensity of emitted light polarized parallel to the excitation light, and *I*_┴_ was the intensity of emitted light polarized perpendicular to the excitation light.

### Cell penetrability assessment

FITC-labeled DEPTAC (100 μM) was dissolved in sterilized water, and administrated into the medium of cultured primary rat hippocampal neurons for 24 h. Neurons were fixed with 4% paraformaldehyde, co-stained with DAPI (Beyotime), and then washed with PBS for 3 × 5 min. FITC was observed under a laser-scanning microscopy (Zeiss).

### CCK8 cell viability assay

HEK293(hTau) cells were administrated with increasing concentration of DEPTAC for 24 h. About 5000 cells were dispensed into each well of a 96-well plate pre-incubated in a humidified incubator under 37 °C, 5% CO_2_. A total volume of 10 μL CCK-8 solution was delivered into each well of the plate. The plate was incubated for 1–4 h, and the light absorbance was measured at 450 nm using a microplate reader (BioTek-Synergy2). Cell viability (V) was evaluated by the formula: *V* = (*A*_d_ – *A*_0_) / (*A*_v_ – *A*_0_) × 100%, where *A*_d_ and *A*_v_ was the absorbance of DEPTAC-treated and vehicle-treated cells, respectively. *A*_0_ was the absorbance of wells with only culture medium and CCK8.

### Brain stereotaxic injection

Mice weighting 20–30 g were anesthetized with 1% pentobarbital sodium (35 mg/kg), and fixed in a stereotaxic instrument (RWD). The scalp was sterilized with iodophors and 75% ethanol, and incised along the skull midline. Holes were drilled. Adeno-associated virus or DEPTAC was injected into the dorsal DG (posterior 1.9 mm, lateral ±1.1 mm from the bregma, ventral −2.0 from the skull) or lateral ventricle (posterior 0.22 mm, lateral ±1.0 mm from the bregma, ventral −2.5 from the skull), respectively, using an automatic microinjection system (World Precision Instruments, USA) at a rate of 0.05 μL/min. The needle syringe was left in place for 5 min before withdrawal. The skin was sutured and sterilized with iodophors. Mice were placed on thermos tank for analepsia.

### In vivo DEPTAC administration

For 2-month male C57BL/6 mice, 10 mM FITC-labeled or unlabeled-DEPTAC were directly injected through the tail vein or lateral ventricle for the evaluation of BBB-penetrability. For 9-month 3×Tg AD mice or Tau368 (20–30 g body weight), unlabeled-DEPTAC (5 mM, 1 μL for each time) was delivered for once into the lateral ventricle through direct stereotaxic injection, or repeatedly administrated through guiding cannulas (RWD, Shenzhen, China) implanted into the lateral ventricle once every 3 days for a consecutive month to test its dephosphorylation efficiency. Mice were restricted in a custom designed device and stayed awake during drug administration, and sacrificed at 24 h post the single or last DEPTAC administration.

### Western blotting and co-immunoprecipitation

Cultured cells or hippocampus tissues were collected on ice, and homogenized with RIPA lysis buffer containing 50 mM Tris-HCl, 100 mM NaCl, 1% Triton X-100, 5 mM EDTA, and 1:100 PMSF, and then centrifuged at 12,000 × *g* for 20 min. The supernatant was collected for the analysis of soluble proteins. In some experiments, the pellets were further incubated with 8% SDS buffer at 37 °C and then ultrasound for 60 times to reach a complete resuspension for the analysis of insoluble tau. Protein concentration was measured through BCA assays (Thermo Fisher). Equal amount of protein from each sample were separated in SDS-PAGE gels, and then transferred onto nitrocellulose or 0.22 μm polyvinylidene fluoride membranes (Merck Millipore). The membranes were blocked with 5% BSA, and incubated in turn with primary and horseradish peroxidase-conjugated secondary antibodies: pS199-Tau (44734G, Invitrogen, 1:2000), pT205-Tau (11108-1, Signalway, 1:2000), pS396-Tau (11102, Signalway, 1:2000), pS404-Tau (11112, Signalway, 1:2000), AT8 (MN1020, Thermo Fisher, 1:1000), Tau5 (ab80579, Abcam, 1:1000), Tau46 (T9450, Sigma, 1:1000), PP2A-Bα (4953, Cell signaling, 1:1000), Phospho-Ser/Thr (ab17464, Abcam1:1000), Ubiquitin (ab7254, Abcam, 1:1000), MAP2 (AB5622, Millipore, 1:2000), hTau-N368 (a generous gift from Prof. Keqiang Ye, Emory University School of Medicine, 1:1000), DEPTAC (developed by Daian Biotechnology, Wuhan, China, 1:500), β-actin (ab6276, Abcam, 1:2000). HRP-conjugated goat anti-rabbit IgG (A0208, Beyotime, 1:3000), HRP-conjugated goat anti-mouse IgG (A0216, Beyotime, 1:3000). Blots were visualized using ECL luminol reagent (P0018FS, Beyotime), and quantified using ImageJ software.

For co-immunoprecipitation, cultured cells were lysed on ice with RIPA buffer for 10 min containing 1:100 PMSF. Protein samples were incubated with protein G agarose (Beyotime) at 4 °C for 2 h, and then centrifuged for 5 min (4 °C, 12,000 rpm). Supernatants were subsequently incubated with specified antibodies and protein G agarose overnight at 4 °C. The agarose beads were washed three times with PBS, and resuspended in 30 μL buffer containing 2% SDS, 100 mM dithiothreitol, 10% glycerol, and 0.25% bromophenol blue, and then denatured at 95 °C for 5 min. Immunoprecipitants were subsequently analyzed through Western blotting.

### Immunostaining

Cultured neurons were fixed with 4% paraformaldehyde for 15 min. For animal experiments, mice were anesthetized with 1% pentobarbital sodium and intracardially perfused with normal saline followed by 4% paraformaldehyde (in 0.1 M phosphate buffer, pH 7.4). Mice brains were removed, post-fixed in 4% PFA for 12 h, and then cryoprotected in 20% and 30% sucrose solutions in turn. Fifty micrometer brain sections were sliced using a cryostat microtome (Leica). Free-floating sections or fixed neurons were washed in PBS, blocked in a buffer containing 5% bull serum albumin and 0.3% Triton x-100 for 1 h, and then incubated with primary antibodies at 4 °C for 12–24 h: tau antibodies (with a 1:200 dilution) were as used in the Western blotting. After washed in PBS, sections or neurons were incubated with fluorescein-conjugated or HRP-conjugated secondary antibodies at 37 °C for 1.5 h (with a 1:500 dilution). For immunofluorescence, sections were mounted with a buffer containing NaHCO_3_ (220.2 mM), Na_2_CO_3_ (28.3 mM), and 50% glycerol. For immunohistochemistry staining, endogenous peroxidase activity was eliminated by incubating brain slices in 0.3% H_2_O_2_ in PBS at 37 °C for 30 min before serum blocking, and immunoreactions were developed using a DAB-staining kit (ZSGB-Bio). Sections were then dehydrated through graded ethanol series, and sealed with neutral balsam. Images were taken by a virtual slide Microscope (SV120, Olympus) or two-photon laser-scanning confocal microscope (LSM710, Zeiss).

### Thioflavin T staining

Free-floating brain sections were washed with Tris-buffered saline (TBS) for 3 × 5 min, and then incubated with 0.3% Thioflavin T (Sigma) dissolved in 50% ethanol at room temperature for 10 min. The sections were then decolorized in 50% ethanol for 3 × 5 min, washed in TBS, and subsequently co-stained with DAPI for 10 min.

### Electron microscopy

Mice were killed by overdose 1% pentobarbital sodium. Hippocampal tissues were isolated on ice, fixed in 2.5% glutaraldehyde at 4 °C for 24 h, washed in 0.1 M phosphate buffer, and post-fixed in 1% osmium acid for 2 h. After washed with phosphate buffer, tissues were dehydrated in increasing gradient of ethanol (30–100%), and incubated in turn with acetone/epoxy (2:1), acetone/epoxy (1:1) and epoxy for 12 h each at 37 °C. Tissues were subsequently embedded in epoxy at 37 °C for 48 h. Sections of 100 nm were sliced using a ultramicrotome (EM UC7, Leica), and stained in a buffer containing 2% uranium acetate and lead citrate at room temperature for 15 min. Images were taken by a transmission electron microscope (Tecnai G^2^ 20 TWIN, FEI). Bundles density and length were analyzed using ImageJ.

### Sparse labeling

A total volume of 0.3 μL mixture of AAV-CaMKIIα-Cre (5.21E+07) and AAV-EF1α-DIO-mCherry (3.24E+12) (1:1 vol/vol) was stereotaxically injected into the dDG of Tau368 mice 1 month following the beginning of dox or normal drinking water administration. Dox was stopped 1-month post AAVs injection, and DEPTAC were delivered through lateral ventricle for a consecutive month. Behavioral tests were performed after the DEPTAC administration, and mice were killed after behavioral tests. Hippocampal sections of 60 μm were obtained, and fluorescent images were taken by a two-photon laser-scanning confocal microscope (LSM710, Zeiss). The dendrite complexity of granular cells in the dorsal dentate gyrus was evaluated by Sholl analysis using *Simple Neurite Tracer* plugin and ImageJ (Fiji) software. Dendrite spine density and the averaged area of mossy fiber punctas were evaluated using ImageJ by an experimenter blinded from mice groupings. It should be noted that the fluorescence-labeled mossy fibers assembled densely in the CA3 subset, the main target area of mossy fiber axon terminals, making it difficult to accurately measure the puncta areas (data not shown). Therefore, we chose the hilus of DG subset for the measurement, which we believe should be also valid to indicate the axon plasticity of DG granular cells.

### Object-place and novel-object recognition tests

Mice were repeatedly handled during DEPTAC administration before behavioral tests. For object-place recognition in the probe phase, mice were placed in a box (50 × 50 × 50 cm) marked with a visual cue on the wall, and two identical objects (plastic toys) were placed at the two different corners. Each mouse was allowed to freely explore for 5 min, and then removed from the box for 2 min, during which the box and the objects were cleaned with 75% ethanol. Pseudorandomly, one of the two objects (object B) was moved to a new corner while the other object (object A) remained unmoved. In the test phase, mice were allowed to freely explore in the box for another 5 min. Videos were recorded and analyzed online using an ANY-maze video tracking system (Stoelting). The time mice stayed around each object was counted as exploring; and the preference ratio was calculated as the exploring time (B − A) / (B + A).

Novel-object recognition tests were performed one day after the object-place recognition. A similar protocol was used except that object B was replaced by a novel object (object C) in the test phase, and mice preference for the novel object was measured.

### Morris water maze test

Morris water maze tests began 3 days after the novel-object recognition tests. Briefly, mice were trained to find a hidden platform submerged under water in a Morris water maze (1.2 m in diameter) for five consecutive days with three trials per day. Visual cues outside the pool remained constant. In each trial, mice were pseudorandomly placed, facing the pool wall, in one of the three quadrants without platform and allowed to freely seek the hidden platform within 60 s. Mice failed to find the target were guided to the platform and placed for another 10 s. The traveling path of each mouse was recorded and analyzed online using an MWZ-100 video-tracking system (Techman, China). At day 6, each mouse was tested for 60 s in the water maze with the hidden platform removed. Videos were recorded using recorded and time of mice stayed in, and number of entries into the target quadrant previously place the platform were analyzed using MWZ-100 and ANY-maze video tracking system (Stoelting).

### Statistical analysis

Data were presented as means ± SEM, unless otherwise specified. All data were analyzed and plotted using SPSS Statistics (IBM) or GraphPad Prism (GraphPad Software). Unpaired two-tailed *t*-tests, one-way, two-way or repeated measures ANOVA followed by Tukey’s multiple comparisons tests were used as illustrated in each figure legend. *p* < 0.05 was considered as statistically significant. Statistical results of all comparisons in this paper were shown in the supplementary material.

## Supplementary information

Supplementary materials

## Data Availability

All data or resources used in the paper are available by reasonable requirements to the leading correspondence, Prof. Jian-Zhi Wang (wangjz@mail.hust.edu.cn).
