# Supplementary material for: A novel dephosphorylation targeting chimera selectively promoting tau removal in tauopathies
Source: Signal Transduct Target Ther. 2021 Jul 14;6:269. doi: 10.1038/s41392-021-00669-2 (PMC8280143; doi:10.1038/s41392-021-00669-2)
Supplement: Supplementary file 1 — Supplementary materials [file 41392_2021_669_MOESM1_ESM.pdf]

Supplementary Materials for

A novel dephosphorylation targeting chimera selectively promoting tau removal in tauopathies

Jie Zheng†*, Na Tian†, Fei Liu, Yidian Zhang, Jingfen Su, Yang Gao, Mingmin Deng, Linyu Wei, Jingwang Ye, Honglian Li, Jian-Zhi Wang*

†These authors contributed equally to this work. Correspondence to: zhengjie@zmu.edu.cn (Jie Zheng), wangjz@mail.hust.edu.cn (Jian-Zhi Wang)

This PDF file includes:

Supplementary Fig. S1-S8

Statistical results of all comparisons in this paper

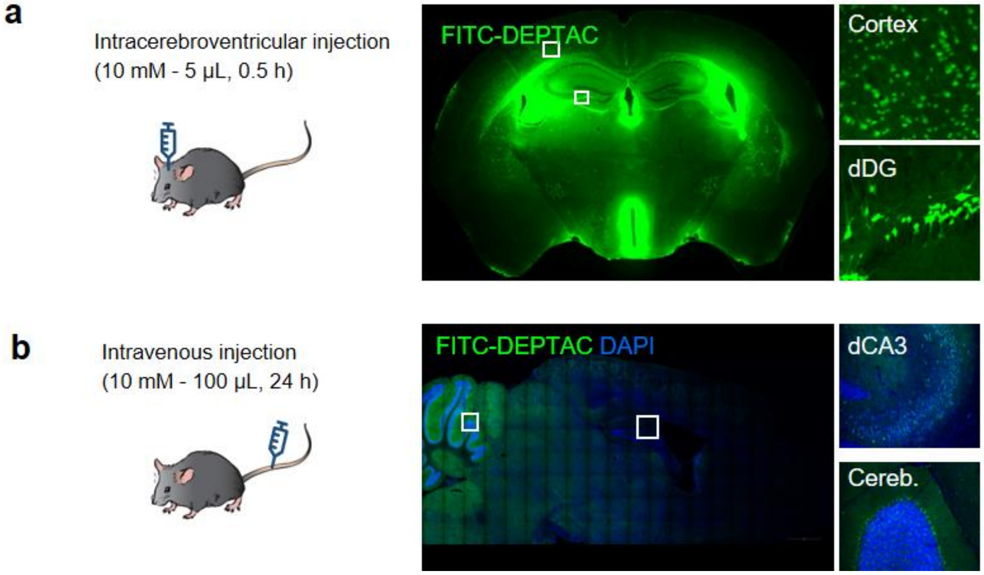

Fig. S1. DEPTAC shows high cell-penetrability *in vivo* with limited trans-BBB efficiency. (**a**) DEPTAC showed high neuron-penetrability when delivered through intracerebroventricular infusion. (**b**) DEPTAC showed limited trans-brain blood barrier (BBB) efficiency when delivered through the mouse tail vein.

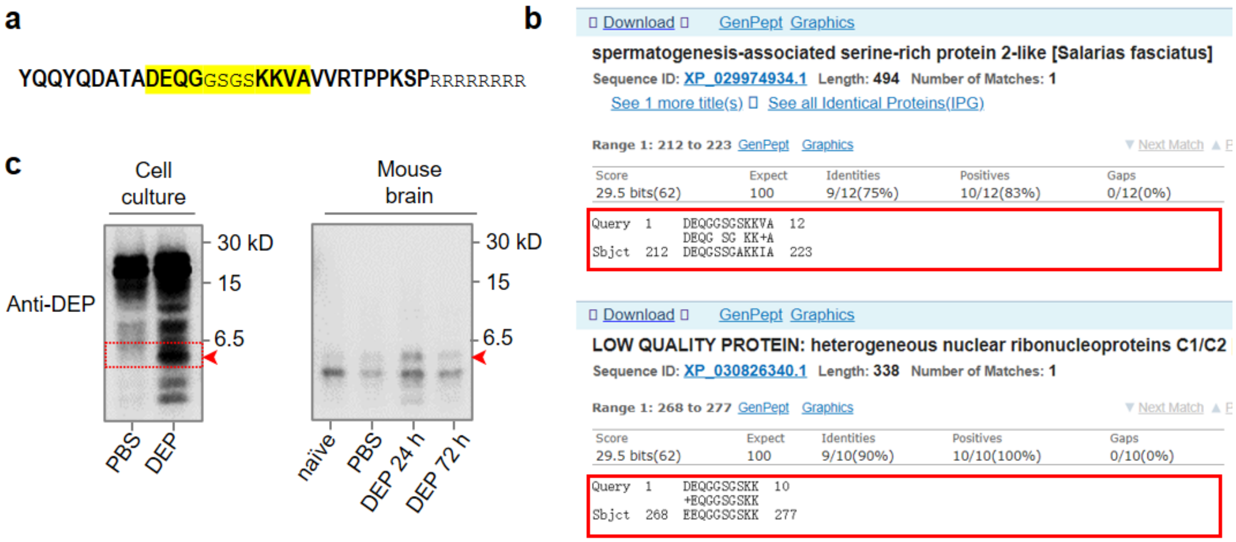

Fig. S2. The DEPTAC antibody. (**a**) A peptide of *DEQGGSGSKKVA* from DEPTAC was synthesized and used for the preparation of rabbit polyclonal DEPTAC antibody. (**b**) The sequence *DEQGGSGSKKVA* shows low homology with existed proteins in NCBI BLAST. Even the top 2 homologous proteins both share less than 9 amino acids, which were generally recognized as the shortest peptide fragment to show enough immunogenicity, with the target sequence DEQGGSGSKK. (**c**) The anti-DEPTAC showed acceptable specificity in recognizing DEPTAC at about 4.8 kD.

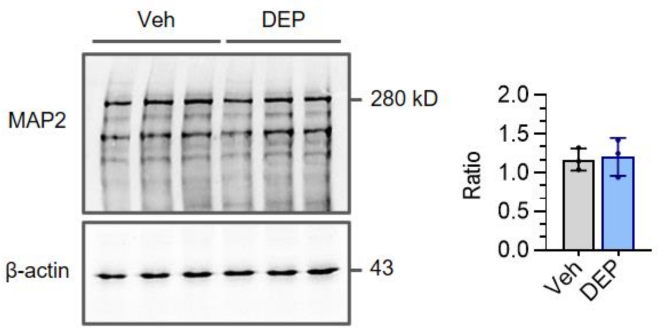

Fig. S3. DEPTAC does not affect MAP2 level. DEPTAC 200 μM for 24 h did not change the level of MAP2 in primary cultured neurons. Unpaired t test.

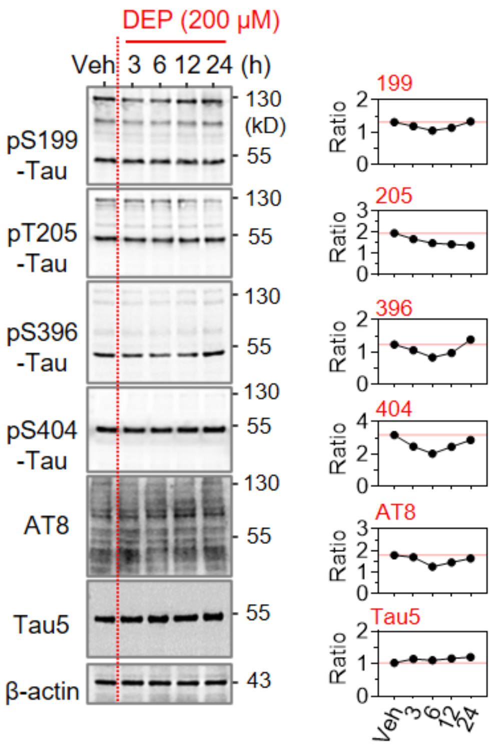

Fig. S4. DEPTAC shows limited effect in dephosphorylating and reducing tau in hTau-expressed HEK293 cells. Samples were collected 24 hours post vehicle or DEPTAC (DEP) administration.

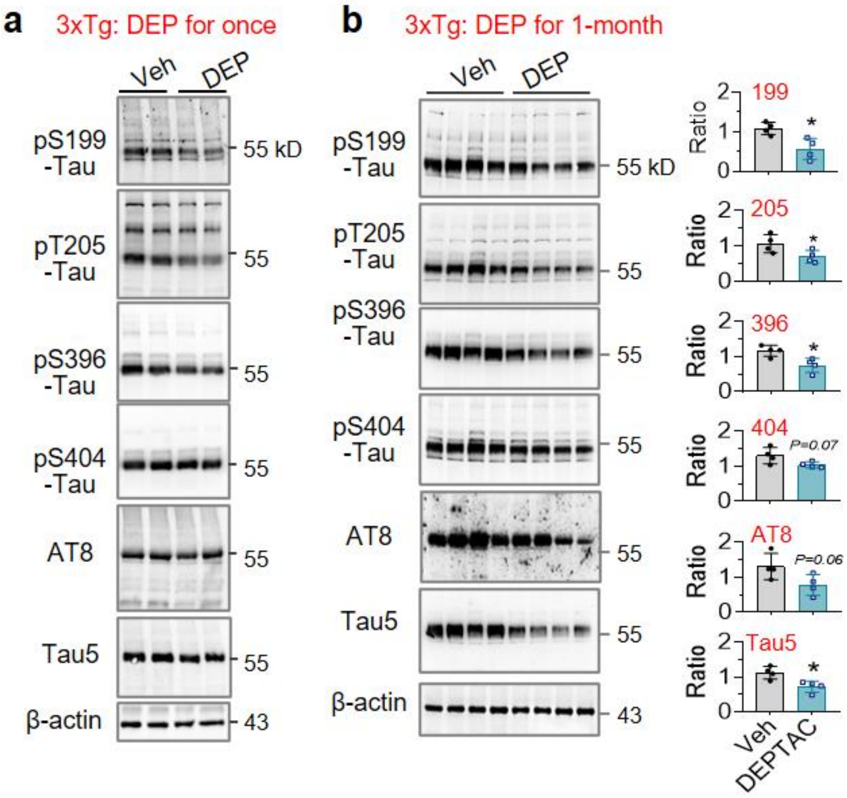

Fig. S5. DEPTAC promotes tau dephosphorylation at multiple sites in 3×Tg AD mice. (**a**) DEPTAC tended to decrease phospho-tau and total tau after delivery for once into the hippocampus of 9-month 3×Tg AD mice. (**b**) Repeated intracerebroventricular administration of DEPTAC for a consecutive month significantly reduced the phospho-tau and total tau in the hippocampus of 9-month 3×Tg AD mice. Unpaired t tests, * $p < 0.05$, n = 4 mice in each group.

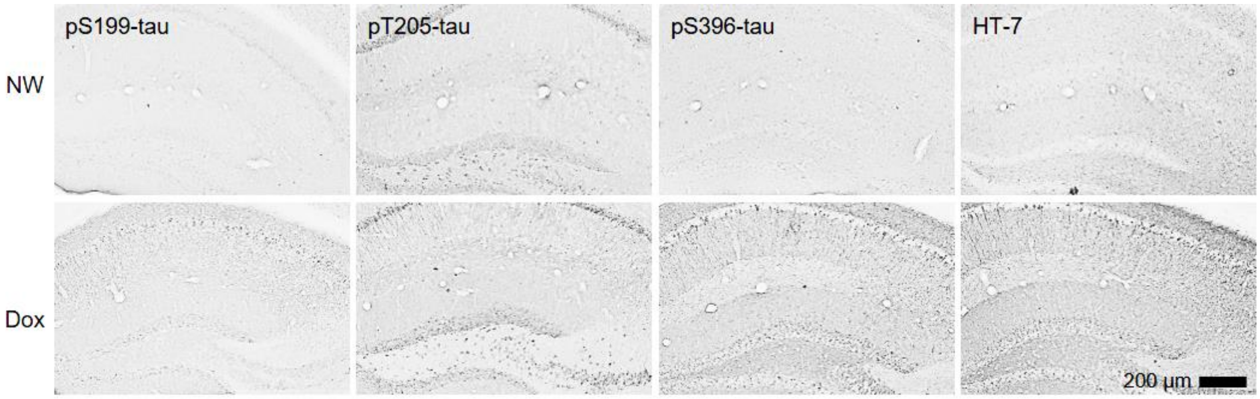

Fig. S6. Dox treatment for one month prominently increases tau phosphorylation in the hippocampus of Tau368 mice. HT-7 antibody recognizes human tau. Scale bar, 200 µm.

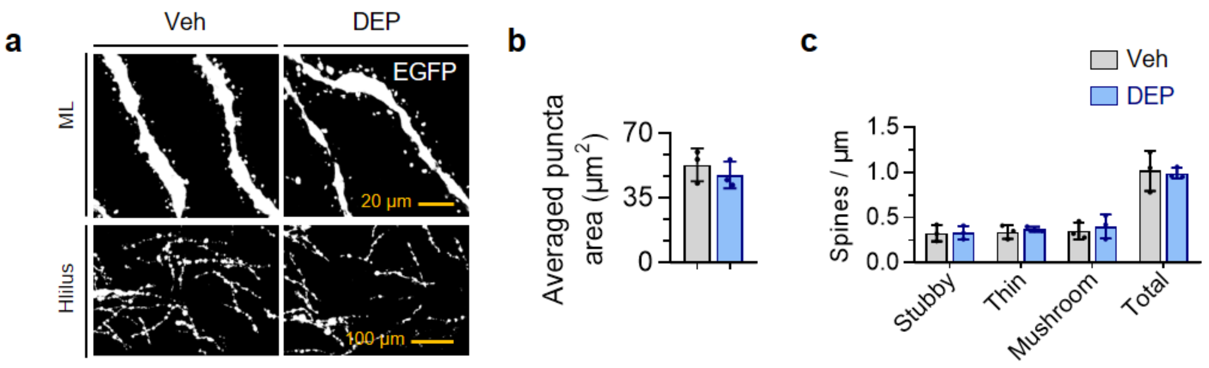

Fig. S7. DEPTAC shows limited effect on neurite plasticity in Tau368 mice without dox treatment. (**a**) Representative images showing the dendrite spines and mossy fiber punctas. (**b, c**) DEPTAC had limited effects on the averaged mossy fiber punctas area (b) and dendritic spine density (c) of dDG granular cells. Unpaired t tests or Two-way ANOVA followed by Tukey's multiple comparisons, n = 3 mice in each group.

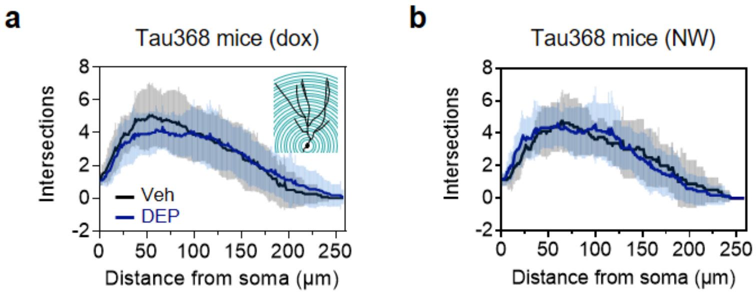

Fig. S8. DEPTAC shows limited effect on dendrite complexity of dentate granular cells. DEPTAC did not statistically change dendrite complexity of DG granular cells in Tau368 mice treated with dox (a) and NW (b). Repeated measures ANOVA, n = 3~5 mice and 12~22 neurons in each group.

Statistical methods and results of all comparisons in this paper

Fig. 1c

DEP & Tau:

Two-way ANOVA followed by Tukey's multiple comparisons tests. Concentration effect: F (7, 32) = 20.19, $P < 0.001$; Peptide effect: F (2, 32) = 164.5, $P < 0.001$; Interaction: F (14, 32) = 10.76, $P < 0.001$. DEP *vs.* Ctrl-peptide, $P < 0.001$; DEP *vs.* FITC, $P < 0.001$; Ctrl-peptide *vs.* FITC, $P < 0.05$.

DEP & PP2A-Bα:

Two-way ANOVA followed by Tukey's multiple comparisons tests. Concentration effect: F (7, 32) =42.04, $P < 0.001$; Peptide effect: F (2, 32) = 192.90, $P < 0.001$; Interaction: F (14, 32) = 17.53, $P < 0.001$. DEP *vs.* Ctrl-peptide, $P < 0.001$; DEP *vs.* FITC, $P < 0.001$; Ctrl-peptide *vs.* FITC, $P = 0.08$.

Fig. 1d

FITC-DEP + Tau:

One-way ANOVA. F (7, 16) = 51.29, $P < 0.001$

FITC-DEP + PP2A-Bα:

One-way ANOVA. F (7, 16) = 44.26, $P < 0.001$

Fig. 1f

One-way ANOVA followed by Tukey's multiple comparisons tests. F (4, 25) = 1.47, $P = 0.24$. $P = 0.26$ for C_0 *vs.* C_{25}, $P = 0.64$ for C_0 *vs.* C_{50}, $P = 0.25$ for C_0 *vs.* C_{100}, $P = 0.11$ for C_0 *vs.* C_{200}.

Fig. 2c

Concentration gradients:

(1) pS199-tau: One-way ANOVA followed by Tukey's multiple comparisons tests. F (5, 12) = 38.81, $P < 0.001$. $P = 0.14$ for C_0 *vs.* C_{25}, $P = 0.31$ for C_0 *vs.* C_{50}, $P = 0.42$ for C_0 *vs.* C_{100}, $P < 0.05$ for C_0 *vs.* C_{200}, $P < 0.01$ for C_0 *vs.* C_{300}.

(2) pT205-tau: One-way ANOVA followed by Tukey's multiple comparisons tests. F (5, 12) = 32.09, $P < 0.001$. $P = 0.31$ for C_0 *vs.* C_{25}, $P = 0.16$ for C_0 *vs.* C_{50}, $P = 0.12$ for C_0 *vs.* C_{100}, $P < 0.05$ for C_0 *vs.* C_{200}, $P < 0.01$ for C_0 *vs.* C_{300}.

(3) pS396-tau: One-way ANOVA followed by Tukey's multiple comparisons tests. F (5, 12) = 22.09, $P < 0.01$. $P = 0.11$ for C_0 *vs.* C_{25}, $P = 0.13$ for C_0 *vs.* C_{50}, $P = 0.19$ for C_0 *vs.* C_{100}, $P = 0.25$ for C_0 *vs.* C_{200}, $P < 0.05$ for C_0 *vs.* C_{300}.

(4) pS404-tau: One-way ANOVA followed by Tukey's multiple comparisons tests. F (5, 12) = 26.18, $P < 0.01$. $P = 0.23$ for C_0 *vs.* C_{25}, $P = 0.20$ for C_0 *vs.* C_{50}, $P = 0.14$ for C_0 *vs.* C_{100}, $P = 0.14$ for C_0 *vs.* C_{200}, $P < 0.05$ for C_0 *vs.* C_{300}.

(5) AT8: One-way ANOVA followed by Tukey's multiple comparisons tests. F (5, 12) = 39.26, $P < 0.001$. $P = 0.31$ for C_0 *vs.* C_{25}, $P = 0.12$ for C_0 *vs.* C_{50}, $P < 0.05$ for C_0 *vs.* C_{100}, $P < 0.05$ for C_0 *vs.* C_{200}, $P < 0.01$ for C_0 *vs.* C_{300}.

(6) Tau5: One-way ANOVA followed by Tukey's multiple comparisons tests. F (5, 12) = 28.10, $P < 0.001$. $P = 0.22$ for C_0 *vs.* C_{25}, $P = 0.34$ for C_0 *vs.* C_{50}, $P = 10.10$ for C_0 *vs.* C_{100}, $P < 0.05$ for C_0 *vs.* C_{200}, $P < 0.01$ for C_0 *vs.* C_{300}.

Time gradients:

(1) pS199-tau: One-way ANOVA followed by Tukey's multiple comparisons tests. F (5, 12) = 23.19, $P < 0.001$. $P < 0.05$ for T_0 *vs.* $T_{0.5}$, $P < 0.05$ for T_0 *vs.* T_1, $P < 0.05$ for T_0 *vs.* T_3, $P = 0.13$ for T_0 *vs.* T_5, $P = 0.28$ for T_0 *vs.* T_7.

(2) pT205-tau: One-way ANOVA followed by Tukey's multiple comparisons tests. F (5, 12) = 18.04, $P < 0.01$. $P < 0.05$ for T_0 *vs.* $T_{0.5}$, $P < 0.05$ for T_0 *vs.* T_1, $P < 0.05$ for T_0 *vs.* T_3, $P = 0.20$ for T_0 *vs.* T_5, $P = 0.17$ for T_0 *vs.* T_7.

(3) pS396-tau: One-way ANOVA followed by Tukey's multiple comparisons tests. F (5, 12) = 20.85, $P < 0.001$. $P < 0.05$ for T_0 *vs.* $T_{0.5}$, $P < 0.05$ for T_0 *vs.* T_1, $P < 0.05$ for T_0 *vs.* T_3, $P = 0.22$ for T_0 *vs.* T_5, $P = 0.13$ for T_0 *vs.* T_7.

(4) pS404-tau: One-way ANOVA followed by Tukey's multiple comparisons tests. F (5, 12) = 28.19, $P < 0.001$. $P < 0.05$ for T_0 *vs.* $T_{0.5}$, $P < 0.05$ for T_0 *vs.* T_1, $P < 0.05$ for T_0 *vs.* T_3, $P = 0.10$ for T_0 *vs.* T_5, $P = 0.18$ for T_0 *vs.* T_7.

(5) AT8: One-way ANOVA followed by Tukey's multiple comparisons tests. F (5, 12) = 8.62, $P < 0.05$. $P = 0.09$ for T_0 *vs.* $T_{0.5}$, $P = 0.15$ for T_0 *vs.* T_1, $P = 0.25$ for T_0 *vs.* T_3, $P = 0.06$ for T_0 *vs.* T_5, $P < 0.05$ for T_0 *vs.* T_7.

(6) Tau5: One-way ANOVA followed by Tukey's multiple comparisons tests. $F (5, 12) = 31.04$, $P < 0.01$. $P < 0.05$ for T_0 *vs.* $T_{0.5}$, $P < 0.05$ for T_0 *vs.* T_1, $P = 0.13$ for T_0 *vs.* T_3, $P = 0.29$ for T_0 *vs.* T_5, $P = 0.68$ for T_0 *vs.* T_7.

Fig. 2d

Two-way ANOVA followed by Tukey's multiple comparisons tests. Epitopes effect: $F (4, 20) =3.03$, $P < 0.005$; DEP effect: $F (1, 20) = 32.69$, $P < 0.001$; Interaction: $F (4, 20) = 0.93$, $P = 0.46$. All $P < 0.05$ for Veh *vs.* DEP in pS199, pT205, pS396, pS404, and AT8.

Fig. 2e

Two-way ANOVA followed by Tukey's multiple comparisons tests. Epitopes effect: $F (6, 42) =39.84$, $P < 0.001$; DEP effect: $F (2, 42) = 39.90$, $P < 0.001$; Interaction: $F (12, 42) = 1.94$, $P = 0.05$.

pS199-tau:

Veh *vs.* DEP, $P < 0.05$; Veh *vs.* DEP+MG132, $P = 0.09$; DEP *vs.* DEP+MG132, $P = 0.21$;

pT205-tau:

Veh *vs.* DEP, $P < 0.01$; Veh *vs.* DEP+MG132, $P < 0.05$; DEP *vs.* DEP+MG132, $P = 0.34$;

pS396-tau:

Veh *vs.* DEP, $P < 0.05$; Veh *vs.* DEP+MG132, $P = 0.08$; DEP *vs.* DEP+MG132, $P = 0.46$;

pS404-tau:

Veh *vs.* DEP, $P < 0.05$; Veh *vs.* DEP+MG132, $P = 0.35$; DEP *vs.* DEP+MG132, $P = 0.41$;

AT8:

Veh *vs.* DEP, $P < 0.05$; Veh *vs.* DEP+MG132, $P < 0.05$; DEP *vs.* DEP+MG132, $P = 0.97$;

Tau5:

Veh *vs.* DEP, $P < 0.05$; Veh *vs.* DEP+MG132, $P = 0.53$; DEP *vs.* DEP+MG132, $P = 0.21$;

Tau46:

Veh *vs.* DEP, $P < 0.05$; Veh *vs.* DEP+MG132, $P = 0.10$; DEP *vs.* DEP+MG132, $P = 0.99$;

Fig. 3g

(1) pS199-tau: Unpaired two-tailed t tests, $t (4) = 3.99$, $P < 0.05$;

(2) pT205-tau: Unpaired two-tailed t tests, $t (4) = 2.84$, $P < 0.05$;

(3) pS396-tau: Unpaired two-tailed t tests, t (4) = 2.99, $P < 0.05$;

(4) pS404-tau: Unpaired two-tailed t tests, t (4) = 5.06, $P < 0.05$;

(5) AT8: Unpaired two-tailed t tests, t (4) = 2.85, $P < 0.05$;

(6) Tau5: Unpaired two-tailed t tests, t (4) = 2.27, $P = 0.05$;

Fig. 3h

(1) pS199-tau: Unpaired two-tailed t tests, t (6) = 0.36, $P = 0.73$;

(2) pT205-tau: Unpaired two-tailed t tests, t (6) = 3.13, $P < 0.05$;

(3) pS396-tau: Unpaired two-tailed t tests, t (6) = 3.32, $P < 0.05$;

(4) pS404-tau: Unpaired two-tailed t tests, t (6) = 3.70, $P < 0.05$;

(5) AT8: Unpaired two-tailed t tests, t (6) = 0.99, $P = 0.35$;

(6) Tau5: Unpaired two-tailed t tests, t (6) =1.82, $P = 0.11$;

Fig. 4c

Two-way ANOVA followed by Tukey's multiple comparisons tests. Epitopes effect: F (5, 24) = 0.03, $P = 0.99$; DEP effect: F (1, 24) = 61.04, $P < 0.001$; Interaction: F (5, 24) = 1.89, $P = 0.13$. All $P < 0.05$ for Veh *vs*. DEP in pS199, pT205, pS396, pS404, AT8 and Tau5.

Fig. 4d

Phospho-tau: Unpaired two-tailed t tests, t (7) =2.49, $P < 0.05$;

ThT: Unpaired two-tailed t tests, t (5) =4.32, $P < 0.01$;

Fig. 4g

Two-way ANOVA followed by Tukey's multiple comparisons tests. Morphology effect: F (3, 32) = 48.33, $P < 0.001$; DEP effect: F (1, 32) = 24.92, $P < 0.001$; Interaction: F (3, 32) = 14.11, $P < 0.001$. $P = 0.25$ for Stubby, $P = 0.06$ for Thin, $P < 0.01$ for Mushroom, $P < 0.001$ for Total.

Fig. 4h

Unpaired two-tailed t tests, t (8) =2.36, $P < 0.05$;

Fig. 5b

Two-way ANOVA followed by Tukey's multiple comparisons tests. Dox effect: F (1, 70) = 24.29, $P < 0.001$; DEP effect: F (1, 70) = 0.20, $P = 0.65$; Interaction: F (1, 70) = 12.09, $P < 0.001$. $P <$

0.05 for NW+Veh *vs.* NW+DEP, *P* < 0.01 for NW+Veh *vs.* Dox+Veh, *P* < 0.05 for Dox+Veh *vs.* Dox+DEP.

Fig. 5c

Two-way ANOVA followed by Tukey's multiple comparisons tests. Dox effect: F (1, 70) = 13.00, *P* < 0.001; DEP effect: F (1, 70) = 0.49, *P* = 0.65; Interaction: F (1, 70) = 6.79, *P* < 0.05. *P* < 0.05 for NW+Veh *vs.* NW+DEP, *P* < 0.01 for NW+Veh *vs.* Dox+Veh, *P* < 0.05 for Dox+Veh *vs.* Dox+DEP.

Fig.6c

Two-way ANOVA followed by Tukey's multiple comparisons tests. Dox effect: F (1, 46) = 2.28, *P* = 0.28; DEP effect: F (1, 46) = 2.30, *P* = 0.15; Interaction: F (1, 46) = 7.06, *P* < 0.05. *P* > 0.05 for NW+Veh *vs.* NW+DEP, *P* < 0.05 for NW+Veh *vs.* Dox+Veh, *P* < 0.05 for Dox+Veh *vs.* Dox+DEP.

Fig.6f

Two-way ANOVA followed by Tukey's multiple comparisons tests. Dox effect: F (1, 46) = 1.62, *P* = 0.43; DEP effect: F (1, 46) = 2.99, *P* = 0.16; Interaction: F (1, 46) = 7.30, *P* < 0.01. *P* > 0.05 for NW+Veh *vs.* NW+DEP, *P* < 0.05 for NW+Veh *vs.* Dox+Veh, *P* < 0.05 for Dox+Veh *vs.* Dox+DEP.

Fig.6g

Repeated measures ANOVA followed by Tukey's multiple comparisons tests. Time effect: F (4, 230) = 50.97, *P* < 0.001; DEP effect: F (3, 230) = 10.31, *P* < 0.001; Interaction: F (12, 230) = 1.17, *P* = 0.31. *P* < 0.05 at day 2, 4 for Dox+Veh *vs.* Dox+DEP.

Fig.6h

Two-way ANOVA followed by Tukey's multiple comparisons tests. Dox effect: F (1, 46) = 11.49, *P* < 0.01; DEP effect: F (1, 46) = 5.51, *P* < 0.05; Interaction: F (1, 46) = 2.12, *P* = 0.15. *P* > 0.05 for NW+Veh *vs.* NW+DEP, *P* < 0.05 for NW+Veh *vs.* Dox+Veh, *P* < 0.05 for Dox+Veh *vs.* Dox+DEP.

Fig.6i

Two-way ANOVA followed by Tukey's multiple comparisons tests. Dox effect: F (1, 46) = 14.99, *P* < 0.01; DEP effect: F (1, 46) = 6.93, *P* < 0.05; Interaction: F (1, 46) = 4.31, *P* < 0.05. *P* > 0.05 for NW+Veh *vs.* NW+DEP, *P* < 0.05 for NW+Veh *vs.* Dox+Veh, *P* < 0.05 for Dox+Veh *vs.* Dox+DEP.

Fig.S3

Unpaired two-tailed t test, t (4) =0.21, *P* = 0.84.

Fig.S4

Statistical analysis was not applied, there was no biological repeat.

	Veh	3 h	6 h	12 h	24 h
pS199-tau / actin	1.32	1.19	1.06	1.15	1.34
pT205-tau / actin	1.96	1.68	1.49	1.43	1.38
pS396-tau / actin	1.24	1.06	0.84	0.98	1.39
pS404-tau / actin	3.19	2.48	2.04	2.46	2.88
AT8 / actin	1.79	1.70	1.26	1.45	1.64
Tau5 / actin	1.03	1.15	1.11	1.16	1.21

Fig.S5b

(1) pS199-tau: Unpaired two-tailed t tests, t (6) = 3.34, *P* < 0.05;

(2) pT205-tau: Unpaired two-tailed t tests, t (6) = 2.35, *P* < 0.05;

(3) pS396-tau: Unpaired two-tailed t tests, t (6) = 3.20, *P* < 0.05;

(4) pS404-tau: Unpaired two-tailed t tests, t (6) = 2.11, *P* = 0.07;

(5) AT8: Unpaired two-tailed t tests, t (6) = 2.21, *P* = 0.06;

(6) Tau5: Unpaired two-tailed t tests, t (6) =3.25, *P* < 0.05;

Fig.S7b

Unpaired two-tailed t tests, t (4) =0.82, *P* = 0.46.

Fig.S7c

Two-way ANOVA followed by Tukey's multiple comparisons tests. Morphology effect: $F (3, 16) = 51.18$, $P < 0.001$; DEP effect: $F (1, 16) = 0.09$, $P = 0.76$; Interaction: $F (3, 16) = 0.12$, $P = 0.96$. $P = 0.07$ for Stubby, $P = 0.33$ for Thin, $P = 0.51$ for Mushroom, $P = 0.29$ for Total.

Fig.S8a

Two-way ANOVA. Distance effect: $F (257, 9287) = 46.28$, $P < 0.001$; DEP effect: $F (1, 9287) = 12.60$, $P = 0.06$; Interaction: $F (257, 9287) = 1.03$, $P = 0.33$.

Fig.S8b

Two-way ANOVA. Distance effect: $F (257, 4643) = 26.95$, $P < 0.001$; DEP effect: $F (1, 4643) = 0.02$, $P = 0.87$; Interaction: $F (257, 4643) = 0.48$, $P > 0.99$.
